# Novel Polyketides Produced by the Endophytic Fungus *Aspergillus Fumigatus* from *Cordyceps Sinensis*

**DOI:** 10.3390/molecules23071709

**Published:** 2018-07-13

**Authors:** Da-Le Guo, Xiao-Hua Li, Dan Feng, Meng-Ying Jin, Yu-Mei Cao, Zhi-Xing Cao, Yu-Cheng Gu, Zhao Geng, Fang Deng, Yun Deng

**Affiliations:** 1 The Ministry of Education Key Laboratory of Standardization of Chinese Herbal Medicine, State Key Laboratory, Breeding Base of Systematic Research Development and Utilization of Chinese Medicine Resources, School of Pharmacy, Chengdu University of Traditional Chinese Medicine, Chengdu 611137, China; guodale@cdutcm.edu.cn (D.-L.G.); 18844144528@163.com (X.-H.L.); 18408210828@163.com (D.F.); ivyweilan@163.com (M.-Y.J.); maycao12@163.com (Y.-M.C.); caozhixing007@163.com (Z.-X.C.); 2 Syngenta Jealott’s Hill International Research Centre, Berkshire RG42 6EY, UK; yucheng.gu@syngenta.com; 3 Sichuan Institute of Food and Drug Control, Chengdu 611731, China; gengzhao713@hotmail.com

**Keywords:** *Aspergillus fumigatus*, *Cordyceps sinensis*, isochromanes, chiral resolution, ECD calculation, cytotoxicity

## Abstract

Five new polyketides, including two pairs of enantiomers and a racemate, were isolated from the fermentation broth of *Aspergillus fumigatus*, an endophytic fungus isolated from *Cordyceps sinensis*. Their structures were identified using one-dimensional (1D) and two-dimensional (2D) NMR experiments, and the absolute configurations of the enantiomers were confirmed using electronic circular dichroism (ECD) calculations. Compounds **1a** and **2a** exhibited inhibitory activity against the MV4-11 cell line in vitro, with IC_50_ values of 23.95 µM and 32.70 µM, respectively.

## 1. Introduction

*Aspergillus fumigatus* (*A. fumigatus*) is an omnipresent saprophytic fungus normally residing in the soil or decaying organic matter [1], and it has the ability to produce secondary metabolites that meet its survival requirements under various environmental conditions [2]. Previous chemical investigations revealed the constituents to be terpenes [3,4], phenolics [5], diketopiperazine [6], and other nitrogen compounds [7,8], which exhibit a variety of biological activities. For instance, fumagillin, which is a typical secondary metabolite from *A. fumigatus*, has the capacity to inhibit angiogenesis in tumor cells [9].

**Figure 1:**
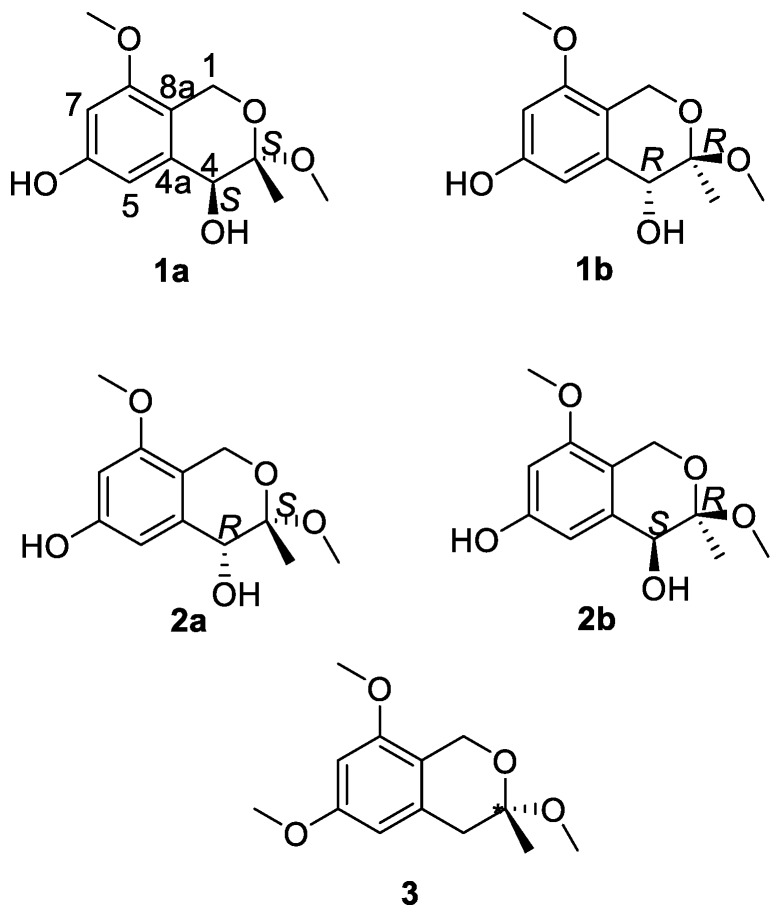
The structures of compounds obtained from *Aspergillus fumigatus*.

With the purpose of searching compounds with novel structures and bio-activities from endophytes of traditional Chinese medicine (TCM), *A. fumigatus*, as an endophytic fungus of *Cordyceps sinensis*, was chosen, and three new isochromanes were isolated from its fermentation broth (Figure 1). These polyketides were presumed to be the mixtures of enantiomers due to their approximate-to-zero optical rotation. Chiral resolution was further applied to two of these racemates, and it yielded two pairs of enantiomers. The absolute configurations of these enantiomers were further verified using quantum-chemical electronic circular dichroism (ECD) calculations. Details of the isolation, structure identification, and cytotoxicity evaluation of these new compounds are reported herein.

## 2. Results and Discussion

**Figure 2:**
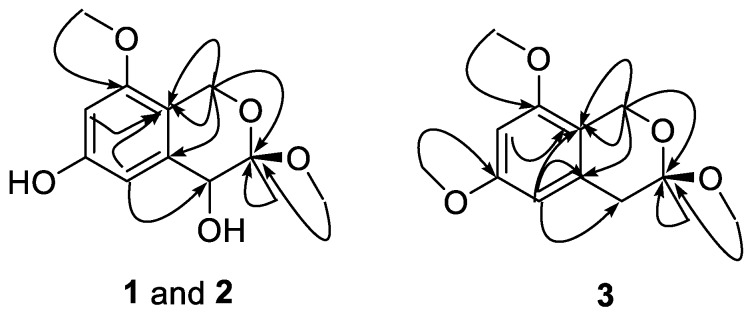
Key HMBC correlations of Compounds **1**–**3**.

**Figure 3:**
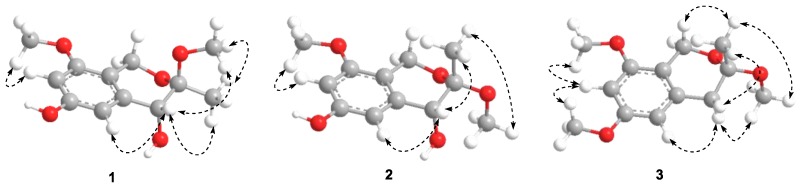
The key NOESY correlations of Compounds **1**–**3**.

Compound **1** (Figure 1) was obtained as a yellow gum. The molecular formula of **1** was established using HRESIMS as C_12_H_16_O_5_ (found 263.0897, calculated for [M + Na]^+^ 263.0890). The infrared (IR) spectrum showed intense absorption bands of hydroxy at ν_max_ 3397.5 cm^−1^ (OH), and 1613.2 cm^−1^ and 1371.6 cm^−1^ (phenyl), as well as a methylene band at 2932.1 cm^−1^. The ^1^H NMR spectrum displayed two aromatic proton signals at *δ* 6.38 (1H, d, *J* = 2.2 Hz, H-5) and 6.33 (1H, d, *J* = 2.2 Hz, H-7), two methoxyl signals at *δ* 3.76 (3H, s, 8-OMe) and 3.30 (3H, s, 3-OMe), one oxymethylene signal at *δ* 4.46 (1H, d, *J* = 15.3 Hz, H-1) and 4.41 (1H, d, *J* = 15.3 Hz, H-1), and one methine signal at *δ* 4.00 (1H, s, H-4) and 1.46 (3H, s, 3-Me). The ^13^C NMR and DEPT spectra of Compound **1** exhibited 12 carbon signals, including six aromatic carbons at *δ* 158.4 (C-8), 157.2 (C-6), 137.3 (C-4a), 114.2 (C-8a), 109.0 (C-5), and 99.0 (C-7), a quaternary carbon at *δ* 101.5 (C-3), one methine carbon at *δ* 70.4 (C-4), one methylene carbon at *δ* 60.7 (C-1), two methoxyl carbons at *δ* 55.8 (8-OMe) and 49.8 (3-OMe), and a methyl signal at *δ* 19.1 (C-3). The HMBC correlations of H-1/C-8 and C-3, H-5/C-4, 3-Me/C-3 and C-4, 3-OMe/C-3, and 8-OMe/C-8, as well as the NOESY correlations of H-7/8-OMe confirmed the presence of an isochromane scaffold, and the primary structure of **1** was established as 3,8-dimethoxy-3-methylisochromane-4,6-diol (Figure 2). The NOESY correlations of H-4/3-OMe indicated that the relative configuration of **1** should be 3R*,4R* (Figure 3). Compound **1** was presumed to be a mixture of enantiomers, as its optical rotation was approximate to zero. Further chiral HPLC analysis confirmed the presence of a pair of anticipated enantiomers. Subsequent chiral resolution was applied, and two enantiomers, **1a** and **1b**, were obtained successfully. The ECD experiment and ECD calculation of **1** were conducted to determine its absolute configuration. The calculated ECD spectra of 3S, 4S-**1** fitted the experimental spectrum of **1a** nicely, while the calculated ECD spectra of 3R, 4R-**1** matched the experimental spectra of **1b** quite well, allowing the absolute configurations of **1a** and **1b** to be determined as 3S, 4S and 3R, 4R, respectively.

**Figure 4:**
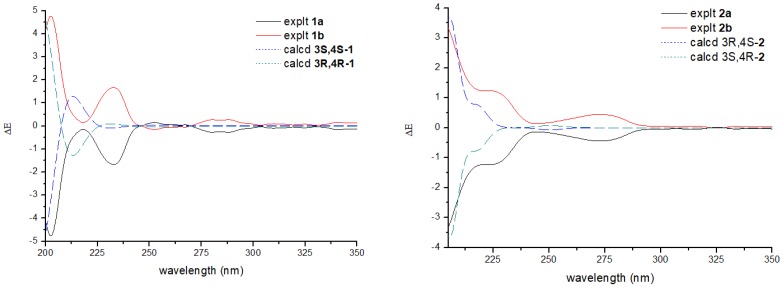
Experimental electronic circular dichroism (ECD) spectra of Compounds **1** and **2** and their calculated curves.

Compound **2** (Figure 1) was obtained as a yellow gum. It was assigned the same molecular formula, C_12_H_16_O_5_, as **1** on the basis of HRESIMS. The NMR spectra of **2** were similar to those of **1**, which indicated that it was an epimer of **1**. Both the changes in chemical shift of H-4 (*δ* 4.36), C-4 (*δ* 72.6), and C-3 (*δ* 99.5), and the absence of NOESY correlation of H-4/3-OMe indicated its relative configuration should be 3R*,4S*. Based on its optical rotation, chiral separation was applied, and it successfully produced a pair of enantiomers. The ECD experiment and ECD calculation of **2b** were conducted to determine its absolute configuration. The results (Figure 4) indicated that the calculated ECD curve of 3R, 4S-**2** was similar to the experimental ECD spectrum of (+)-**2** (**2b**), which designated the configuration of (+)-**2** as 3R, 4S-3,8-dimethoxy-3-methylisochromane-4,6-diol. On the other hand, (−)-**2** was assigned to be 3S, 4R-3,8-dimethoxy-3-methylisochromane-4,6-diol (**2a**) accordingly.

Compound **3** (Figure 1) was isolated as a yellow gum. Its molecular formula was established as C_13_H_18_O_4_ on the basis of the HRESIMS through the pseudo-molecular ion peak at *m*/*z* 261.1107 [M + Na]^+^ (calculated for 261.1097). The ^1^H and ^13^C NMR spectra indicated that **3** possessed a similar structure to **1** and **2** except for a methylene (*δ*_H_: 2.85, d, *J* = 16.4 Hz, 2.73, d, *J* = 16.4 Hz; *δ*_C_: 39.8) instead of a methine group at C-4, and the presence of another methoxyl (*δ*_H_: 3.78; *δ*_C_: 55.7). The HMBC correlation of 6-Me/C-6 readily located this methoxyl at C-6. Thus, **3** was elucidated to be 3,6,8-trimethoxy-3-methylisochromane.

The MTT method was applied to evaluate the cytotoxicity of these compounds against MDA-ME-231 and MV4-11 cancer cell lines. Compounds **1a** and **2a** showed moderate growth inhibition against the MV4-11 cell line with IC_50_ values of 23.95 µM and 32.70 µM, respectively.

## 3. Materials and Methods 

### 3.1. General Experimental Procedures

The UV spectra were measured on a PerkinElmer Lambda 35 UV-VIS spectrophotometer (PerkinElmer, Waltham, MA, USA). The IR spectra were recorded on a PerkinElmer Spectrum One Fourier-transform IR (FT-IR) spectrometer, (PerkinElmer, Waltham, MA, USA). The ECD spectra were obtained on a JASCO (Oklahoma City, OK, USA) J-810 spectrometer. The optical rotations were measured on a JASCO (Oklahoma City, OK, USA) P-1020 polarimeter. The NMR spectra were recorded on a Bruker (Billerica, MA, USA) 400 spectrometer, for one-dimensional (1D) and two-dimensional (2D) NMR. The HRESIMS data were recorded on a Bruker (Billerica, MA, USA) Micro TOF-Q II mass spectrometer. Preparative HPLC was performed on a Hanbon Sci. & Tech. (Huaian, Jiangsu, China) NP7000 serials instrument equipped with a Hanbon Sci. & Tech. (Huaian, Jiangsu, China) NU3000 serials UV detector, using a Kromasil 100-5-C_18_ column (10 × 250 mm, 5μm; Akzo Nobel Pulp and Performance Chemicals AB, Bohus, Sweden) for normal separation, and a column Chiralpak IC column (4.6 × 250 mm, 5 μm; Chiral Technologies, West Chester, PA, USA) for chiral resolution. Column chromatography (CC) was performed on a silica gel (200–300 mesh; Qingdao Marine Chemical Inc., Qingdao, China) and a Sephadex LH-20 (GE-Healthcare Bio-Sciences AB, Uppsala, Sweden). All solvents used were of analytical grade.

### 3.2. Fungal Material 

The *A. fumigatus* strain was separated from *Cordyceps sinensis* collected in Xiahe county, China in May 2017. The fungus was identified using morphological observation and sequence (GenBank acccession No. MG519287) analyses of the ITS region of recombinant DNA (rDNA). The identified strain was inoculated into 24,500 mL Erlenmeyer flasks, each containing 200 mL of potato dextrose agar (PDA) at room temperature, agitated on an orbital shaker at 200 rpm for seven days to produce the seed culture. The fermentation was carried out in one hundred and twenty 1000 mL Fernbach flasks, each containing 10 mL of seed culture and 400 mL of medium (soluble starch 0.8%, peptone 0.5%, NaCl 0.2%, CaCO_3_ 0.2%, MgSO_4_∙7H_2_O 0.05%, and K_2_HPO_4_ 0.05%), and was incubated at 25 °C on a rotary shaker at 200 rpm for 15 days. 

### 3.3. Fractionation and Isolation

The culture was filtered to separate the mycelia and the broth. The broth was firstly extracted with petroleum ether, followed by ethyl acetate. The ethyl acetate solution was concentrated to a brown residue (9.3 g). The crude extract was fractionated using liquid chromatography on a silica gel (6 × 30 cm) with a gradient elution of CHCl_3_/MeOH. The fractions eluted with a ratio of 75:25 were combined, (1.08 g) and were further subjected to Sephadex LH-20 column chromatography (4 × 180 cm; mobile phase CHCl_3_/MeOH, 1:1), which afforded three sub-fractions (Fr.1–Fr.3). Fr.3 (91 mg) was purified using a preparative HPLC with a reversed-phase column (5u 100 A; 10 × 250 mm; mobile phase MeOH/H_2_O, 65:35) to furnish Compounds **1** (3.0 mg), **2** (1.9 mg), and **3** (2.9 mg). Compound **1** was further separated using HPLC with a Chiralpak IC column (mobile phase n-hexane/isopropanol, 85:15) to yield Compounds **1a** (0.33 mg, R_t_ 10.4 min) and **1b** (0.42 mg, R_t_ 20.6 min), while Compound **2** was further separated under the same conditions to give Compounds **2a** (0.16 mg, R_t_ 8.1 min) and **2b** (0.26 mg, R_t_ 28.5 min). 

#### 3.3.1. 3R,4S-3,8-Dimethoxy-3-methylisochromane-4,6-diol (**1a**)

Yellow gum, ${\left[\alpha \right]}_{D}^{20}$ = +167.9 (c = 0.02, MeOH), IR (KBr): 3397.5, 2932.1, 1613.2, 1371.6, 1055.3 cm^−1^; λ_max_ 207.3 (3.74), 281.5 (2.72); ^1^H (CD_3_OD, 400 MHz) and ^13^C NMR (CD_3_OD, 100 MHz) spectroscopic data (see Table 1 and Supplementary Material); HRESIMS *m*/*z* 263.0897 [M + Na]^+^ (calculated for C_12_H_16_O_5_Na^+^, 263.0890).

#### 3.3.2. 3R,4R-3,8-Dimethoxy-3-methylisochromane-4,6-diol (**1b**)

Yellow gum, ${\left[\alpha \right]}_{D}^{20}$ = −167.9 (c = 0.01, MeOH), spectrometric (UV, IR, NMR, MS, and HRESIMS) data are the same as those of **1a**.

#### 3.3.3. 3S,4R-3,8-Dimethoxy-3-methylisochromane-4,6-diol (**2a**)

Yellow gum, ${\left[\alpha \right]}_{D}^{20}$ = −260.1 (c = 0.01, MeOH), IR (KBr): 3421.1, 2940.8, 1623.6, 1384.6, 1061.2 cm^−1^; λ_max_ 211.1 (3.76), 282.2 (2.73); ^1^H (CD_3_OD, 400 MHz) and ^13^C NMR (CD_3_OD, 100 MHz) spectroscopic data (see Table 1); HRESIMS *m*/*z* 263.0893 [M + Na]^+^ (calculated for C_12_H_15_O_5_Na^+^, 263.0890).

#### 3.3.4. 3R,4S-3,8-Dimethoxy-3-methylisochromane-4,6-diol (**2b**)

Yellow gum, ${\left[\alpha \right]}_{D}^{20}$ = +260.1 (c = 0.01, MeOH), spectrometric (UV, IR, NMR, MS, and HRESIMS) data are the same as those of **2a**.

#### 3.3.5. 3,6,8-Trimethoxy-3-methylisochromane (**3**)

Yellow gum, ${\left[\alpha \right]}_{D}^{20}$ = +0.9 (c = 0.11, MeOH), IR (KBr): 3435.5, 2923.0, 1638.5, 1384.2, 1074.7 cm^−1^; λ_max_ 210.6 (3.85), 281.0 (2.97); ^1^H (CD_3_OD, 400 MHz) and ^13^C NMR (CD_3_OD, 100 MHz) spectroscopic data (see Table 1); HRESIMS *m*/*z* 261.1107 [M + Na]^+^ (calculated for C_12_H_16_O_4_Na^+^, 261.1097).

### 3.4. MTT Assay

The MV4-11 and MDA-ME-231 cells (American Type Culture Collection, Manassas, VA, USA) were grown in DMEM or IMDM medium containing 10% FBS in 5% CO_2_ at 37 °C. When the cells entered the exponential growth phase, they were seeded in a 96-well plate and incubated overnight. Afterward, media containing various concentrations of tested compounds from 3.125 µM to 100 µM were added to each well. Additionally, 0.1% DMSO was used as a blank control, while taxol was used as a positive control. After the incubation period (72 h at 37 °C), 20 μL/well of MTT reagent (5 mg/mL) was added, and the wells were incubated for 2–4 h, before 50 μL/well of 20 acidified SDS was added to lyse the cells. Finally, the absorbance was measured at 570 nm to evaluate the inhibition effects of the tested compounds on cell growth. All experiments were performed in triplicate.

## 4. Conclusions

Among the five new compounds obtained from a TCM-related strain *A. fumigatus*, two pairs of novel enantiomers were discovered. The MTT method was used to detect the cytotoxicity of these compounds against MDA-ME-231 and MV4-11 cells. Compounds **1a** and **2a** exhibited moderate cytotoxic activity against the MV4-11 cell line with IC_50_ values of 23.95 µM and 32.70 µM, respectively.

## Figures and Tables

**Table 1:** The ^1^H (400 MHz) and ^13^C (100 MHz) NMR data (*δ* in ppm, multiple *J* in Hz) of Compounds **1**–**3**.

Position	1 ^a^		2		3	
	*δ*_H_	*δ*_C_	*δ*_H_	*δ*_C_	*δ*_H_	*δ*_C_
1	4.41, d, 15.3 Hz 4.46, d, 15.3 Hz	60.7	4.56, d, 15.2 Hz 4.43, d, 15.2 Hz	60.6	4.65, d, 15.1 Hz 4.44, d, 15.1 Hz	60.2
2	-	-	-	-	-	-
3	-	101.5	-	99.5	-	98.8
4	4.00, s	70.4	4.36, s	72.6	2.85, d, 16.4 Hz 2.73, d, 16.4 Hz	39.8
4a	-	137.3	-	138.6	-	134.4
5	6.38, d, 2.2 Hz	109.0	6.63, d, 2.1 Hz	105.5	6.33, d, 2.1 Hz	105.5
6	-	157.2	-	158.5	-	161.0
7	6.33, d, 2.2 Hz	99.0	6.28, d, 2.1 Hz	97.9	6.33 d, 2.1 Hz	96.9
8	-	158.4	-	156.8	-	157.4
8a	-	114.2	-	114.6	-	115.2
3-Me	1.46, s	19.1	1.49, s	20.5	1.44, s	23.4
3-OMe	3.30, s	49.8	3.31, s	49.1	3.28, s	48.8
6-OMe	-	-	-	-	3.78	55.7
8-OMe	3.76, s	55.8	3.75, s	55.8	3.72	55.8

^a^ Compounds **1**–**3** were measured in CD_3_OD.

## Supplementary Materials

The following are available online, Pages 1–3: The computational details; Figures S1–S2: HPLC chromatograms of **1** and **2** using a Chiralpak IC column; Figures S3–S23: MS and NMR spectra of Compounds **1**–**3**.
