# Supplementary material for: Novel Polyketides Produced by the Endophytic Fungus Aspergillus Fumigatus from Cordyceps Sinensis
Source: Molecules. 2018 Jul 13;23(7):1709. doi: 10.3390/molecules23071709 (PMC6100219; doi:10.3390/molecules23071709)
Supplement: Supplementary file 1 [file molecules-23-01709-s001.pdf]

SUPPLEMENTARY MATERIAL

Novel Polyketides Produced by the Endophytic Fungus Aspergillus fumigatus from Cordyceps sinensis

Da-Le Guo[a], Xiao-Hua Li[a], Dan Feng[a], Meng-Ying Jin[a], Yu-Mei Cao[a], Zhi-Xing Cao[a], Yu-Cheng Gu[b], Zhao Geng[c], Fang Deng[a,*] and Yun Deng[a,*]

a. The Ministry of Education Key Laboratory of Standardization of Chinese Herbal Medicine, State Key Laboratory, Breeding Base of Systematic Research Development and Utilization of Chinese Medicine Resources,School of Pharmacy, Chengdu University of Traditional Chinese Medicine, Chengdu 611137, P R China

b. *Syngenta Jealott' s Hill International Research Centre, Berkshire RG42 6EY, UK*

c. *Sichuan Institute of Food and Drug Control,Chengdu, 611731, P R China*

(Tel/Fax: +86 028 61800232. E-mail: dengf99@163.com (Fang DENG), dengyun2000@hotmail.com (Yun DENG))

Abstract: Five new polyketides, including two pairs of enantiomers and a racemate were isolated from the fermentation broth of *Aspergillus fumigatus*, an endophytic fungus isolated from *Cordyceps sinensis*. Their structures were identified by 1D and 2D NMR experiments and the absolute configurations of those enantiomers were confirmed by electronic circular dichroism (ECD) calculations. Compounds 1a, and 2a exhibited inhibitory activity against MV4-11 cell line in vitro with IC50 values of 23.95 μM and 32.70 μM, respectively.

Computational details

The theoretical calculations of compounds **1-2** were performed using Gaussian 03. Conformational analysis was initially carried out using Accelrys Discovery Studio 2.5 to generate conformations by Boltzman Jump, then minimize them by Smart Minimizer using the MMFF molecular mechanics force field. All geometries (17 low energy conformers for (3R, 4R)-**1**, and 21 low energy conformers for (3R, 4S)-**2**, respectively) with relative energy from 0-10.0 kcal/mol were used in optimizations at the B3LYP/6-31G(d) in the gas phase. Room-temperature equilibrium populations were calculated according to the Boltzmann distribution law. The theoretical calculation of ECD was performed using TDDFT at the B3LYP/6-31G (d, p) level in the methanol. The ECD spectra were obtained by weighing the Boltzmann distribution rate of each geometric conformation. SpecDis 1.6 was used to sum up single CD spectra after a Boltzmann statistical weighting, and for the Gauss curve generation and for the comparison with experimental data.

References:

(1) Gaussian 03, revision D.01, M. J. Frisch, G. W. Trucks, H. B. Schlegel, G. E. Scuseria, M. A. Robb, J. R. Cheeseman, J. J. A. Montgomery, T. Vreven, K. N. Kudin, J. C. Burant, J. M. Millam, S. S. Iyengar, J. Tomasi, V. Barone, B. Mennucci, M. Cossi, G. Scalmani, N. Rega, G. A. Petersson, H. Nakatsuji, M. Hada, M. Ehara, K. Toyota, R. Fukuda, J. Hasegawa, M. Ishida, T. Nakajima, Y. Honda, O. Kitao, H. Nakai, M. Klene, X. Li, J. E. Knox, H. P. Hratchian, J. B. Cross, V. Bakken, C. Adamo, J. Jaramillo, R. Gomperts, R. E. Stratmann, O. Yazyev, A. J. Austin, R. Cammi, C. Pomelli, J. W. Ochterski, P. Y. Ayala, K. Morokuma, G. A. Voth, P. Salvador, J. J. Dannenberg, V. G. Zakrzewski, S. Dapprich, A. D. Daniels, M. C. Strain, O. Farkas, D. K. Malick, A. D. Rabuck, K. Raghavachari, J. B. Foresman, J. V. Ortiz, Q. Cui, A. G. Baboul, S. Clifford, J. Cioslowski, B. B. Stefanov, G. Liu, A. Liashenko, P. Piskorz, I. Komaromi, R. L. Martin, D. J. Fox, T. Keith, M. A. Al-Laham, C. Y. Peng, A. Nanayakkara, M. Challacombe, P. M. W. Gill, B. Johnson, W. Chen, M. W. Wong, C. Gonzalez, J. A. Pople, Gaussian, Inc., Wallingford CT, 2004.

(2) T. Bruhn, A. Schaumlöffel, Y. Hemberger, G. Bringmann, SpecDis version 1.61, University of Wuerzburg, Germany, 2013.

(3) Su, B. N.; Park, E. J.; Mbwambo, Z. H.; Santarsiero, B. D.; Mesecar, A. D.; Fong, H. H.; Pezzuto, J. M.; Kinghorn, A. D., New chemical constituents of *Euphorbia quinquecostata* and absolute configuration assignment by a convenient Mosher ester procedure carried out in NMR tubes. J Nat Prod 2002, 65 (9), 1278- 82.

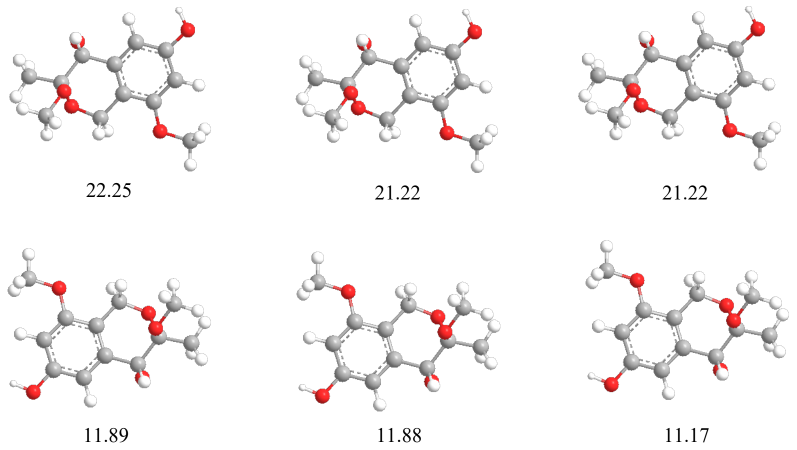

Figure CS1. Most stable conformers of (3R, 4R)-**1** calculated with DFT at the B3LYP/6-31G (d) level. Relative populations are shown below each conformer. Equilibrium Populations calculated by the relative free Gibbs energies at B3LYP/6-31G (d) level in the gas phase, assuming Boltzman statistics at T = 298.15 K and 1 atm.

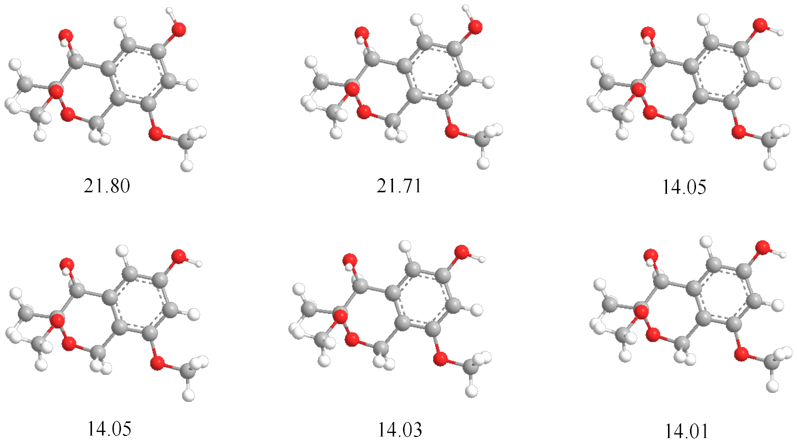

Figure CS2. Most stable conformers of (3R, 4S)-**2** calculated with DFT at the B3LYP/6-31G (d)

level. Relative populations are shown below each conformer. Equilibrium Populations calculated by the relative free Gibbs energies at B3LYP/6-31G (d) level in the gas phase, assuming Boltzman statistics at T = 298.15 K and 1 atm.

Tables of Contents

S1. UV detection of the HPLC chiral separation of racemic **1**

S2. Experimental ECD spectra of **1a** and **1b**, and calculated ECD spectra of 3*R*,4*R*-**1** and 3*S,4S*-**1** in MeOH (UV correction=4nm, band width s=0.3eV).

S3. UV detection of the HPLC chiral separation of racemic **2**

S4. Experimental ECD spectra of **2a** and **2b**, and calculated ECD spectra of 3*S,4R*-**2** and 3*R,4S*-**2** in MeOH (UV correction=4nm, band width s=0.3eV).

S5. HRESIMS spectrum of **1**

S6. ^{1}H NMR spectrum (400 MHz, CD_3OD) of **1**

S7. ^{13}C NMR spectrum (100 MHz, CD_3OD) of **1**

S8. ^{1}H-^{1}H COSY spectrum (CD_3OD) of **1**

S9. HSQC spectrum (CD_3OD) of **1**

S10. HMBC spectrum (CD_3OD) of **1**

S11. NOESY spectrum (CD_3OD) of **1**

S12. HRESIMS spectrum of **2**

S13. ^{1}H NMR spectrum (400 MHz, CD_3OD) of **2**

S14. ^{13}C NMR spectrum (400 MHz, CD_3OD) of **2**

S15.^{1}H-^{1}H COSY spectrum (CD_3OD) of **2**

S16. HSQC spectrum (CD_3OD) of **2**

S17. HMBC spectrum (CD_3OD) of **2**

S18. NOESY spectrum (CD_3OD) of **2**

S19. HRESIMS spectrum of **3**

S20. ^{1}H NMR spectrum (400 MHz, CD_3OD) of **3**

S21. ^{13}C NMR spectrum (100 MHz, CD_3OD) of **3**

S22. ^{1}H-^{1}H COSY spectrum (CD_3OD) of **3**

S23. HSQC spectrum (CD_3OD) of **3**

S24. HMBC spectrum (CD_3OD) of **3**

S25. NOESY spectrum (CD_3OD) of **3**

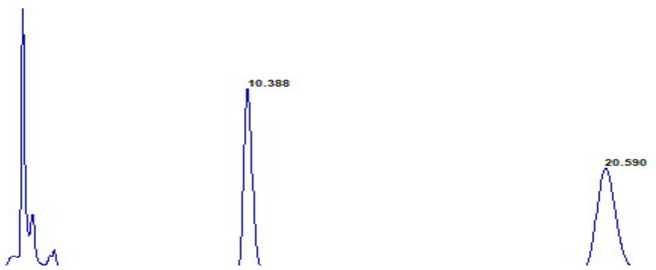

S1. UV detection of the HPLC chiral separation of racemic **1**

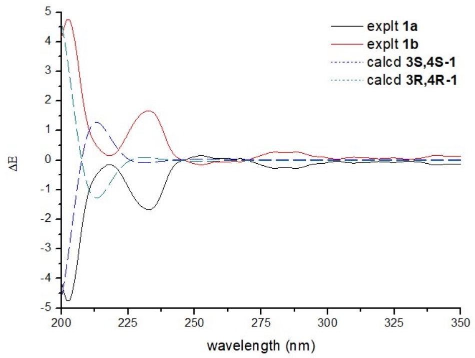

S2. Experimental ECD spectra of **1a** and **1b**, and calculated ECD spectra of 3*R*,4*R*-**1** and 3*S*,4*S*-**1** in MeOH (UV correction=4nm, band width s=0.3eV).

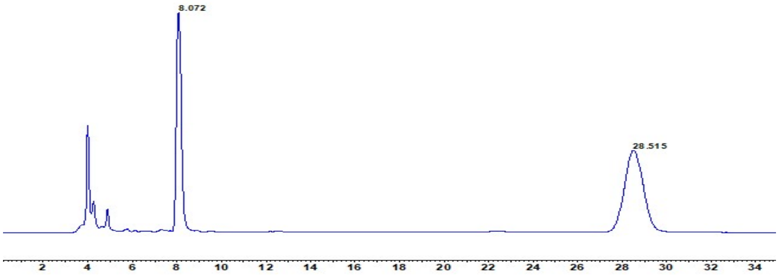

S3. UV detection of the HPLC chiral separation of racemic **2**

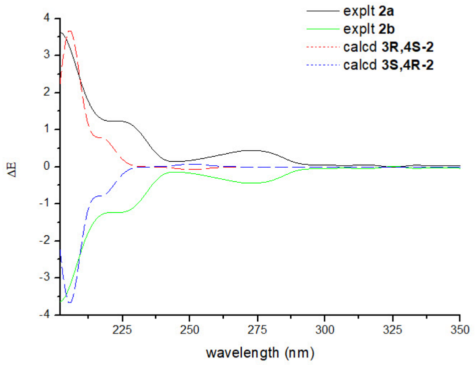

S4. Experimental ECD spectra of **2a** and **2b**, and calculated ECD spectra of 3*R*,4*S*-**2** and 3*S*,4*R*-**2** in MeOH (UV correction=4nm, band width s=0.3eV).

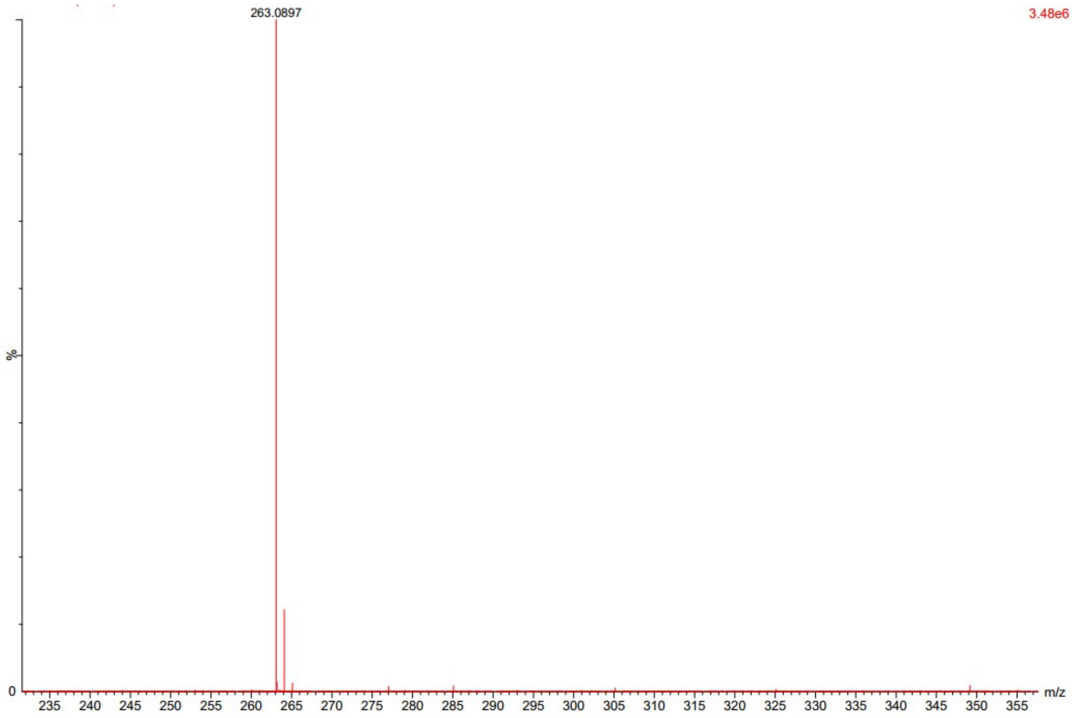

S5. HRESIMS spectrum of **1**

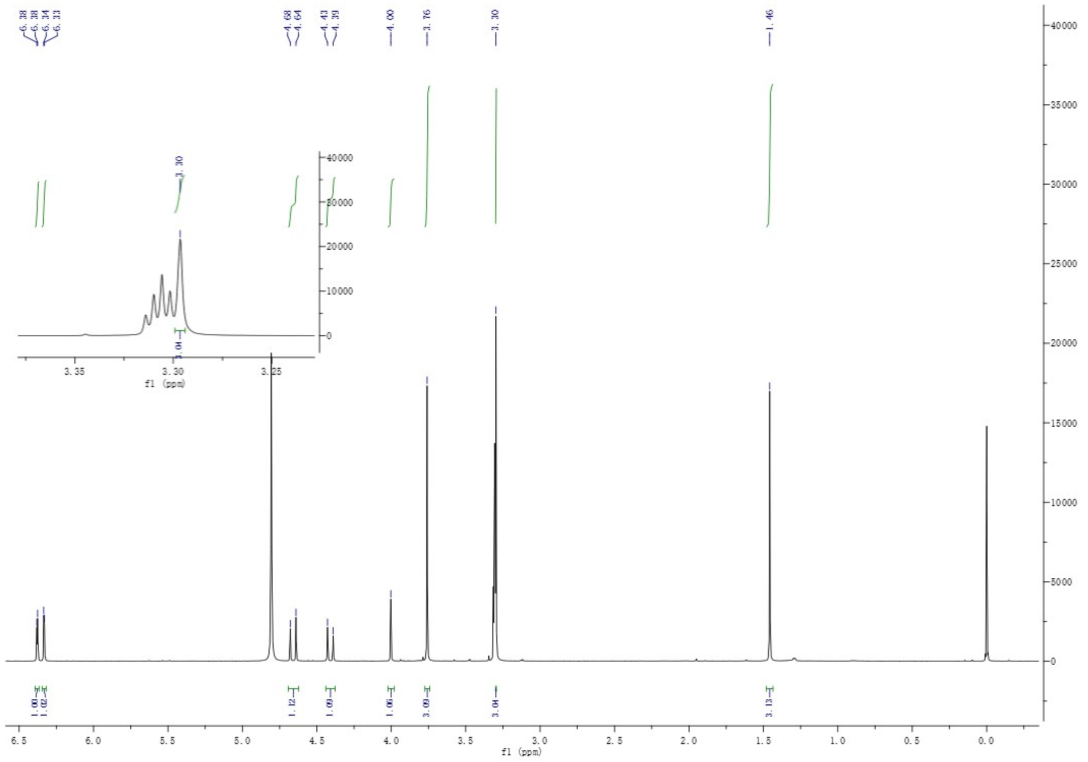

S6. ^{1}H NMR spectrum (400 MHz, CD_3OD) of **1**

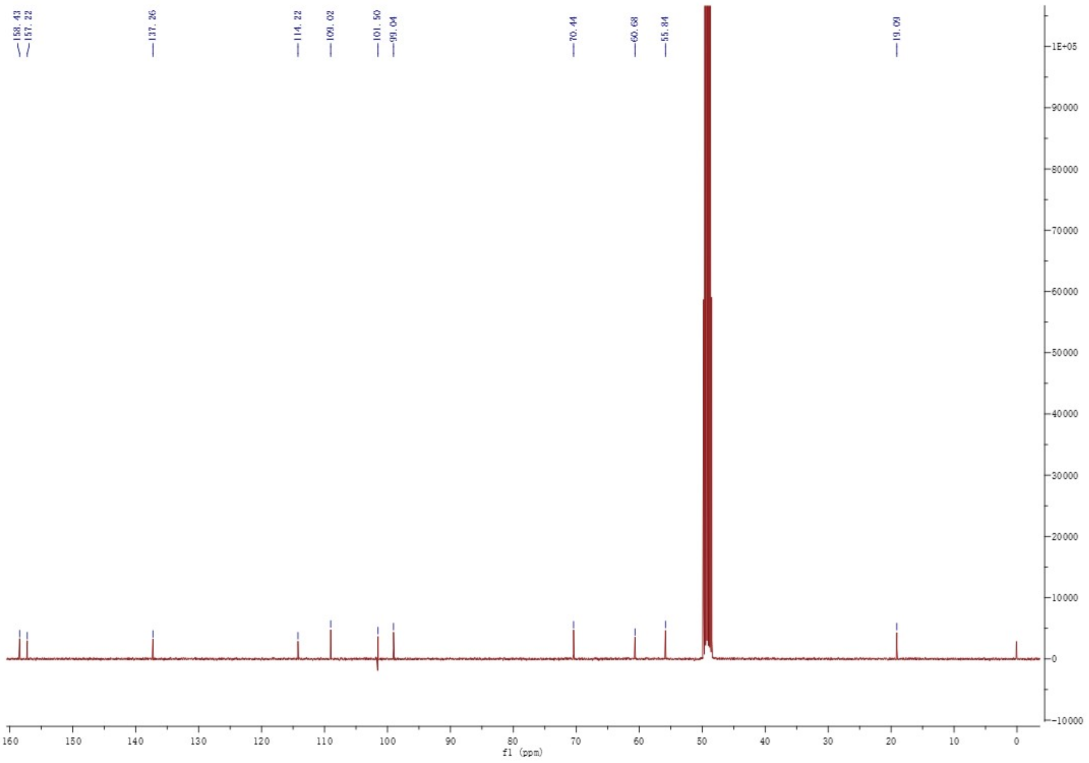

S7. ^{13}C NMR spectrum (100 MHz, CD_3OD) of **1**

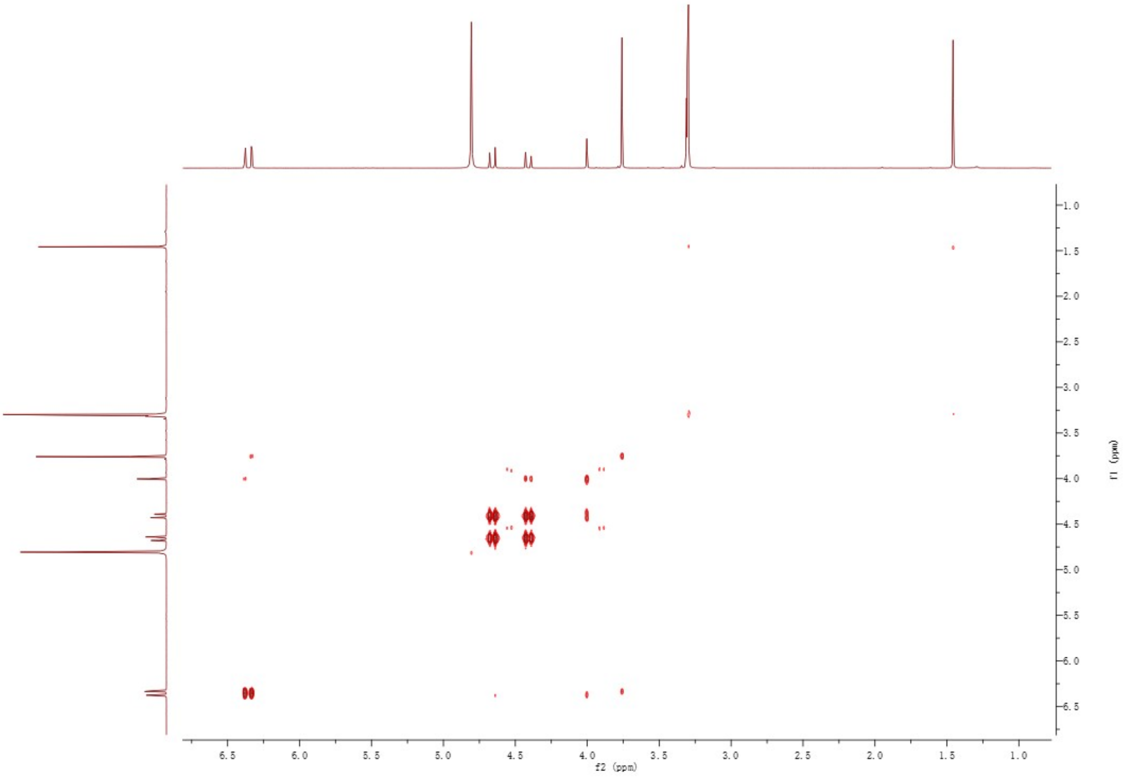

S8. ^{1}H-^{1}H COSY spectrum (CD_3OD) of **1**

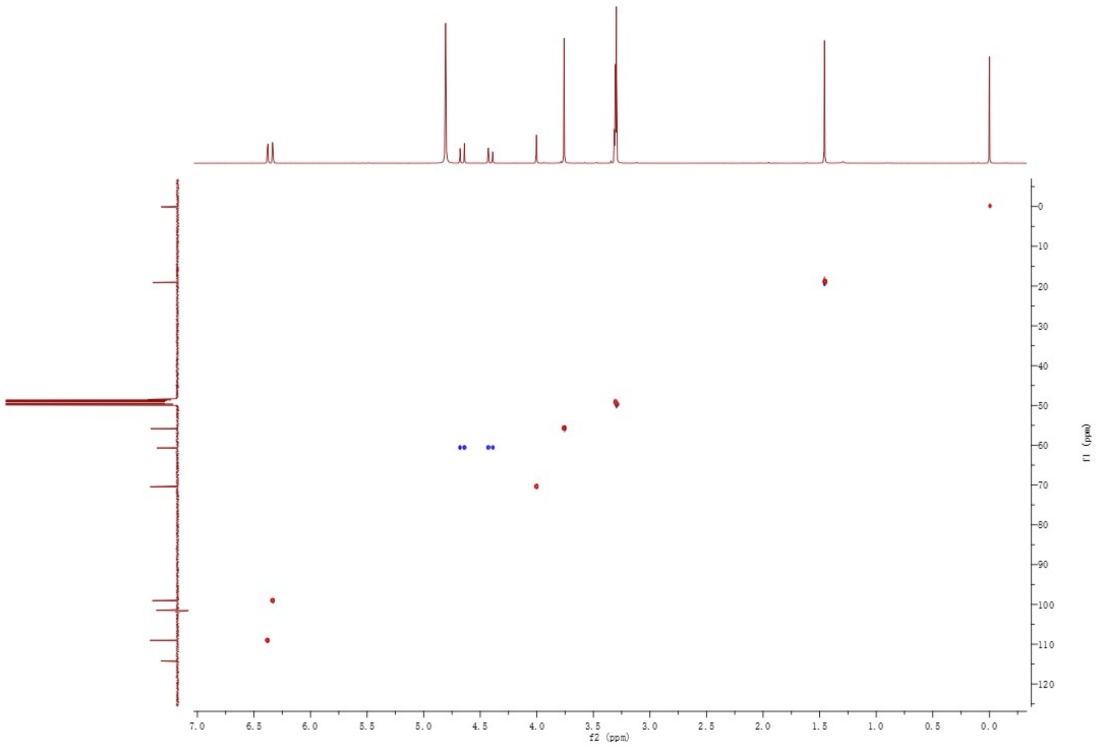

S9. HSQC spectrum (CD_3OD) of **1**

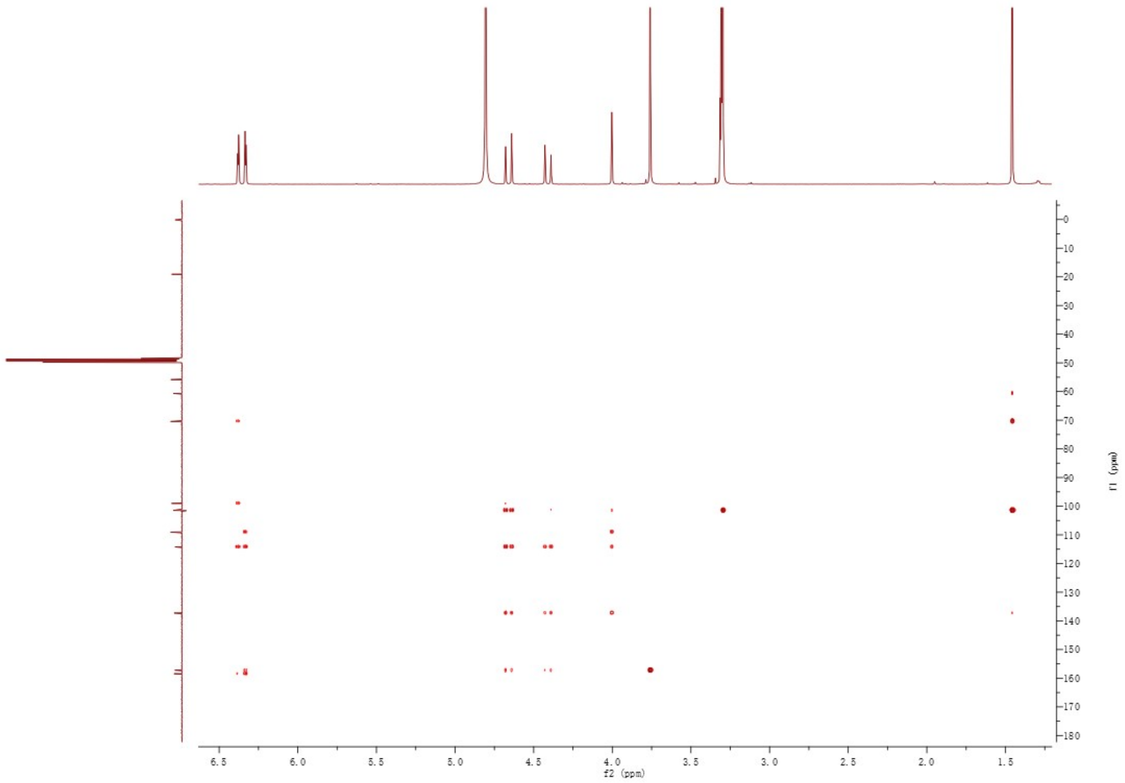

S10. HMBC spectrum (CD_3OD) of **1**

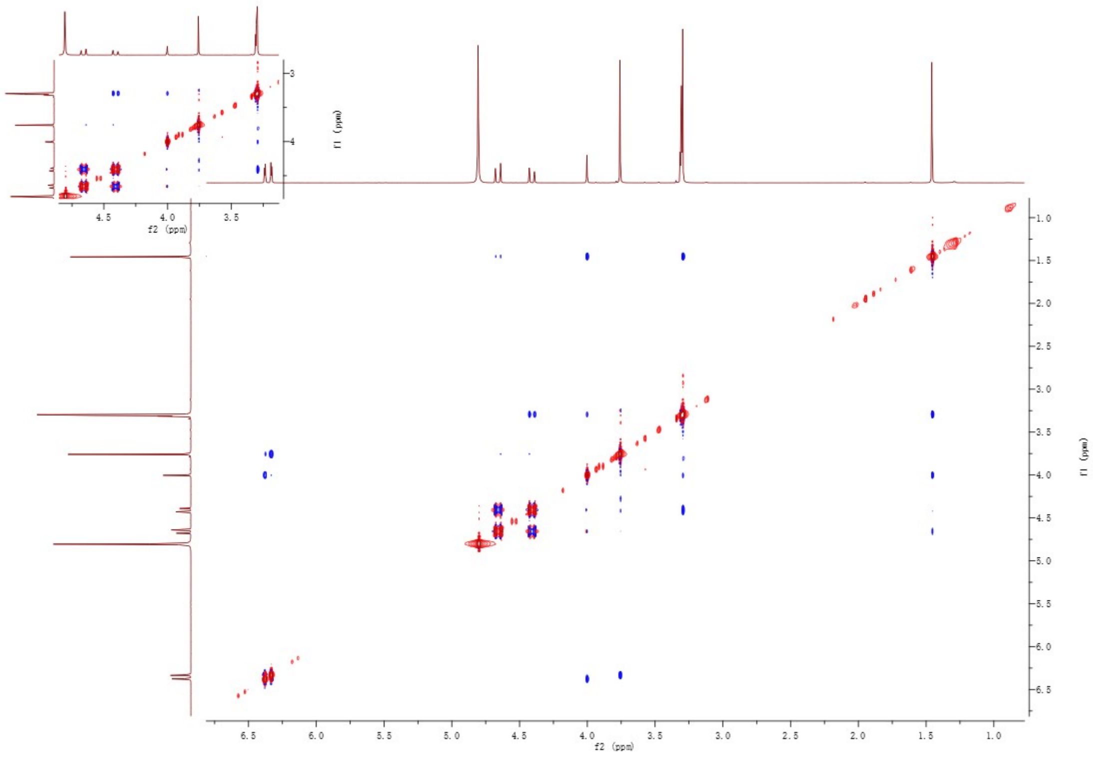

S11. NOESY spectrum (CD_3OD) of **1**

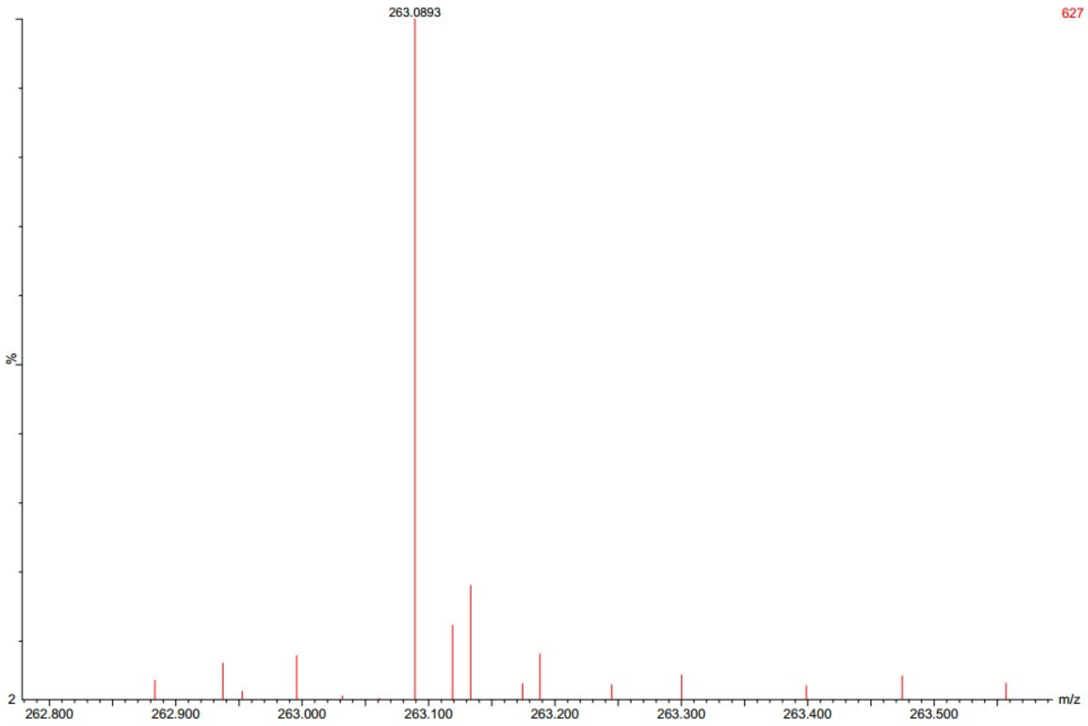

S12. HRESIMS spectrum of **2**

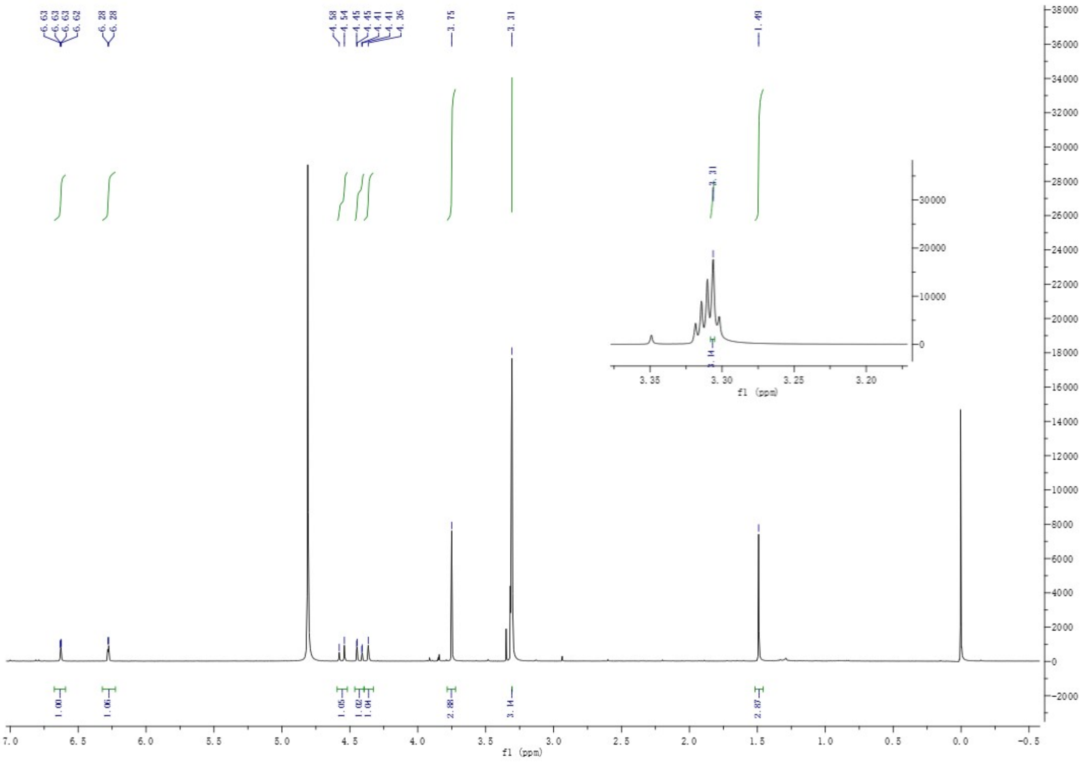

S13. ^{1}H NMR spectrum (400 MHz, CD_3OD) of **2**

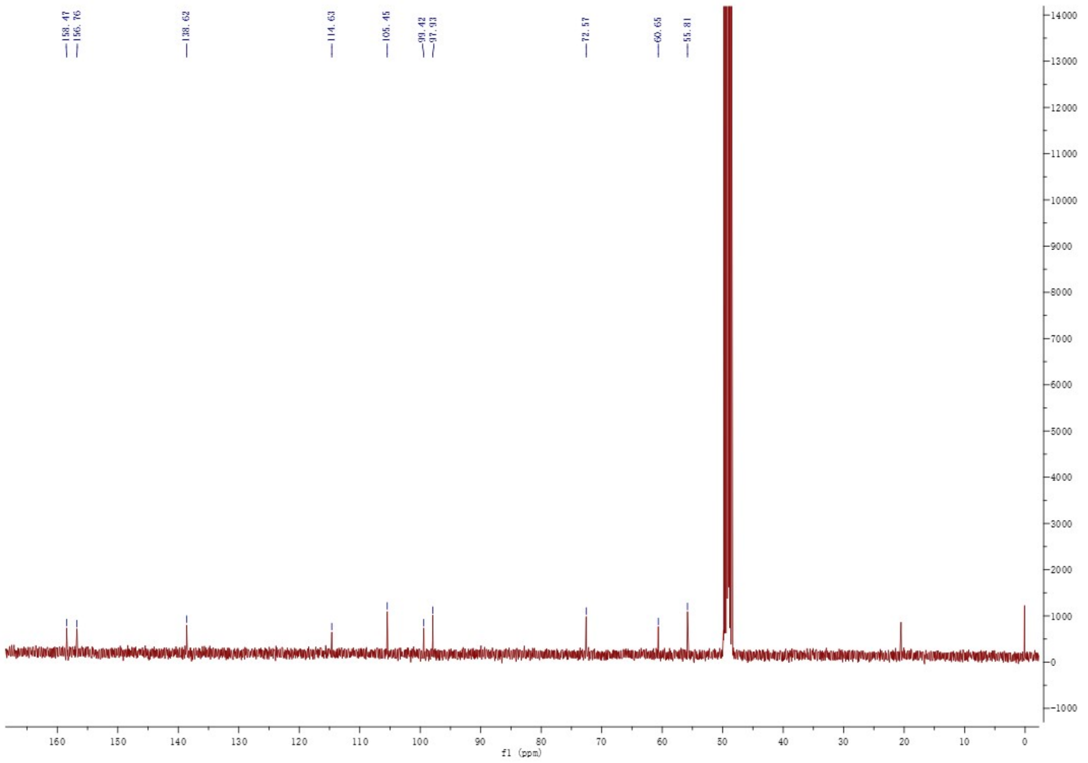

S14. ^{13}C NMR spectrum (400 MHz, CD_3OD) of **2**

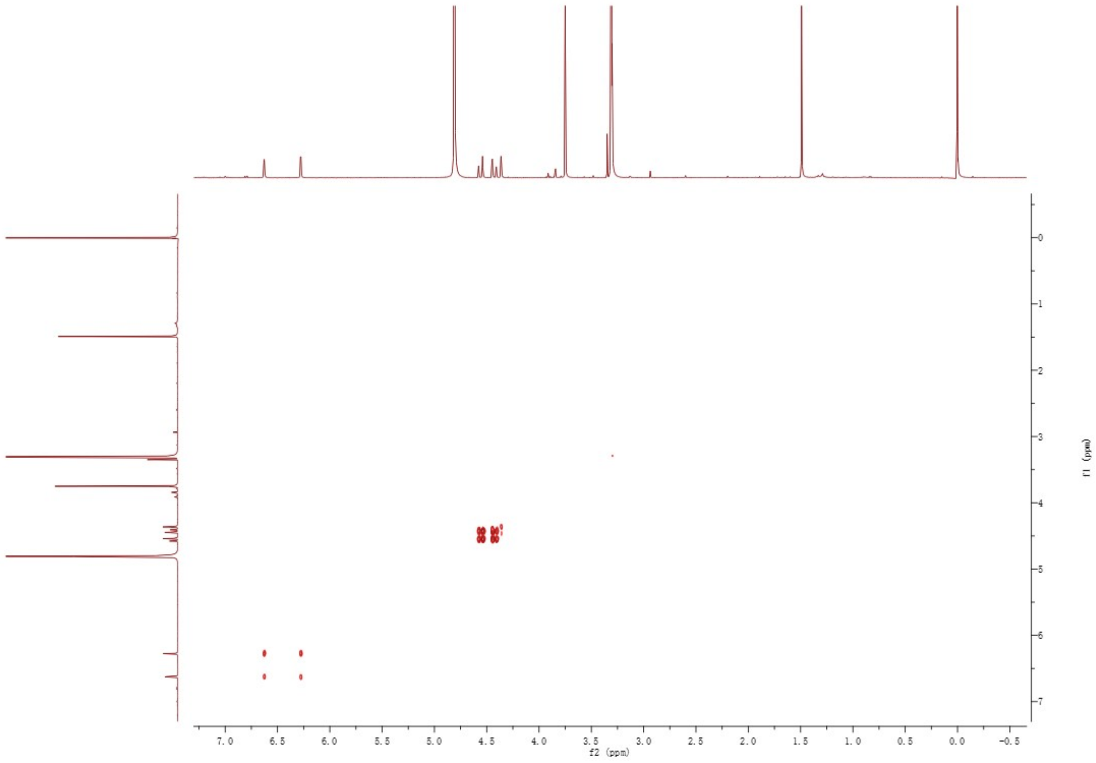

S15.^{1}H-^{1}H COSY spectrum (CD_3OD) of **2**

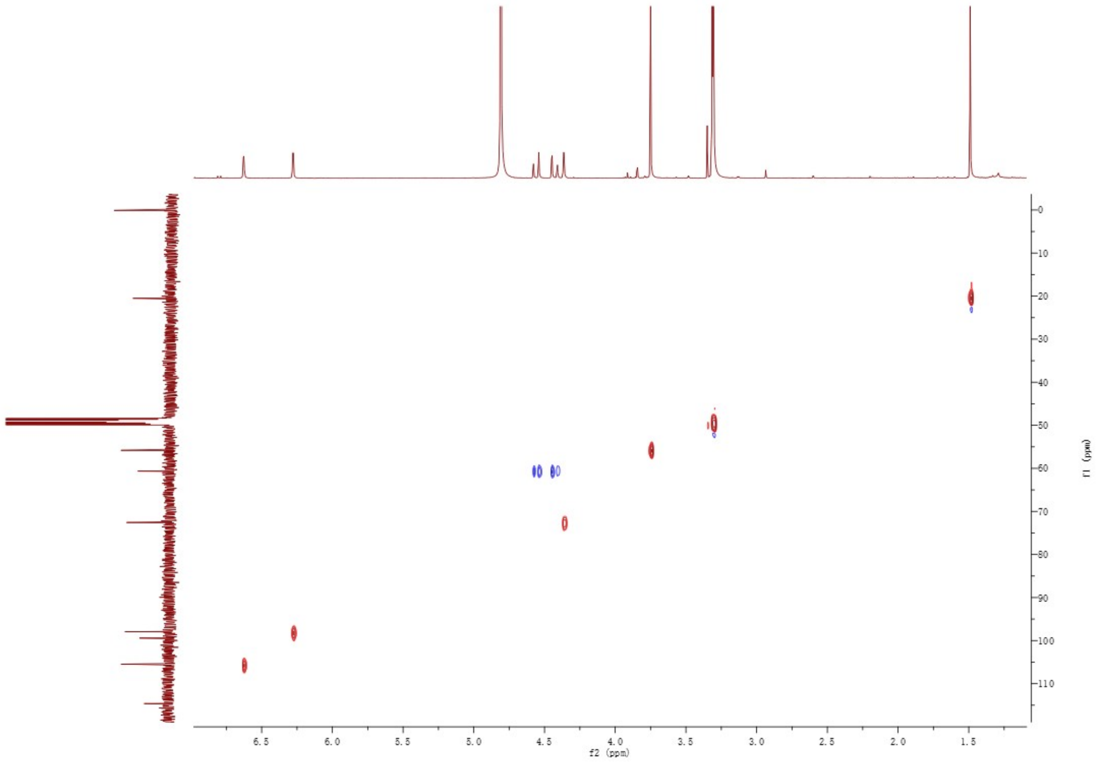

S16. HSQC spectrum (CD_3OD) of **2**

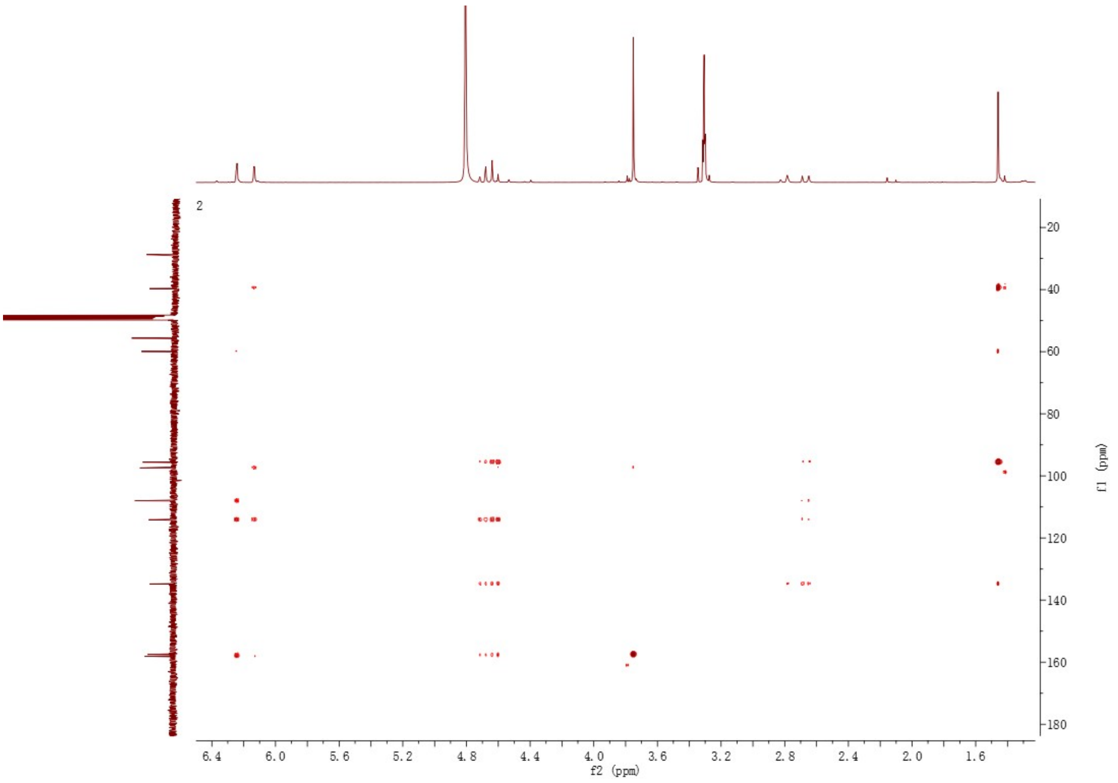

S17. HMBC spectrum (CD_3OD) of **2**

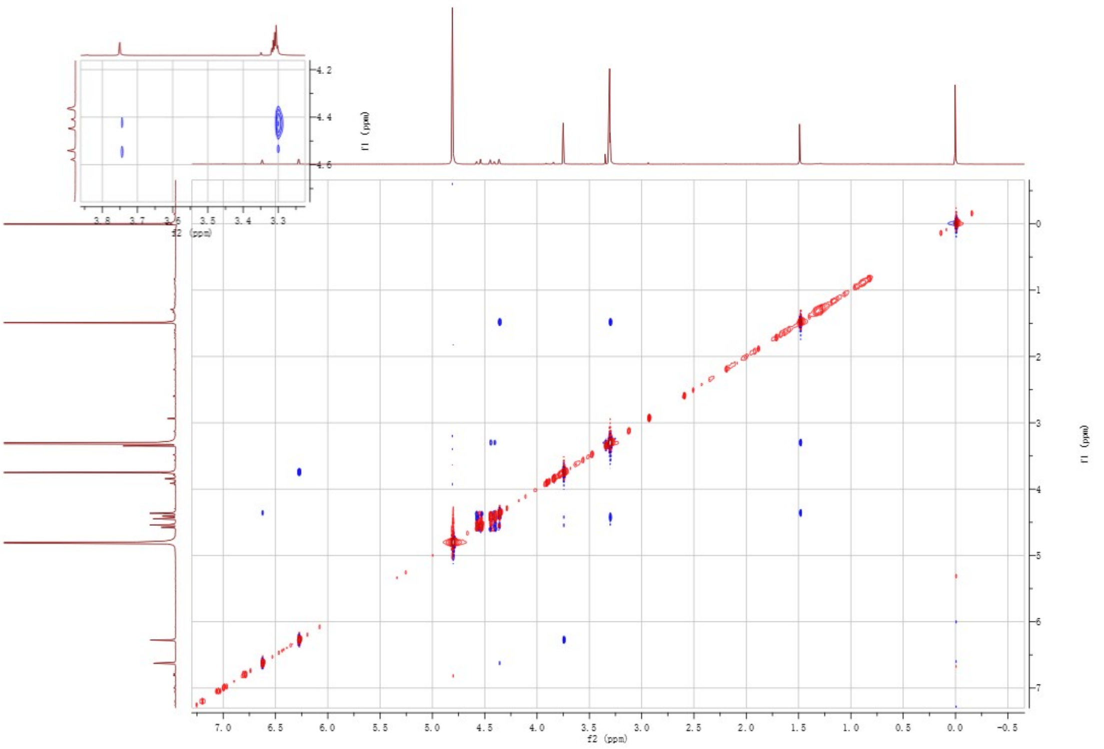

S18. NOESY spectrum (CD_3OD) of **2**

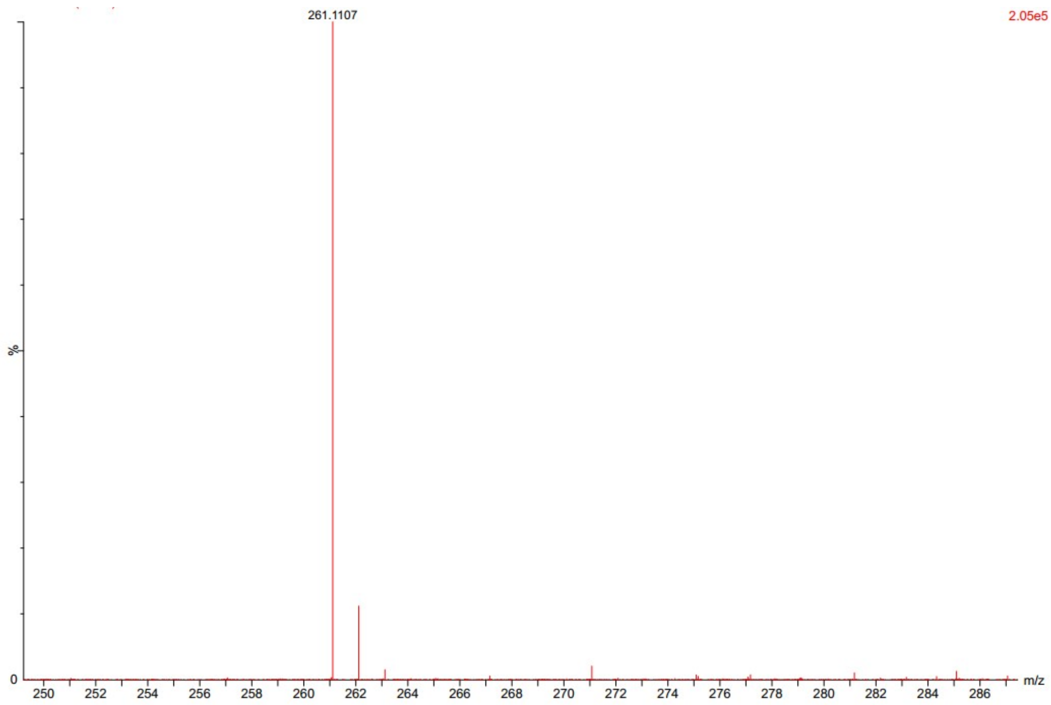

S19. HRESIMS spectrum of **3**

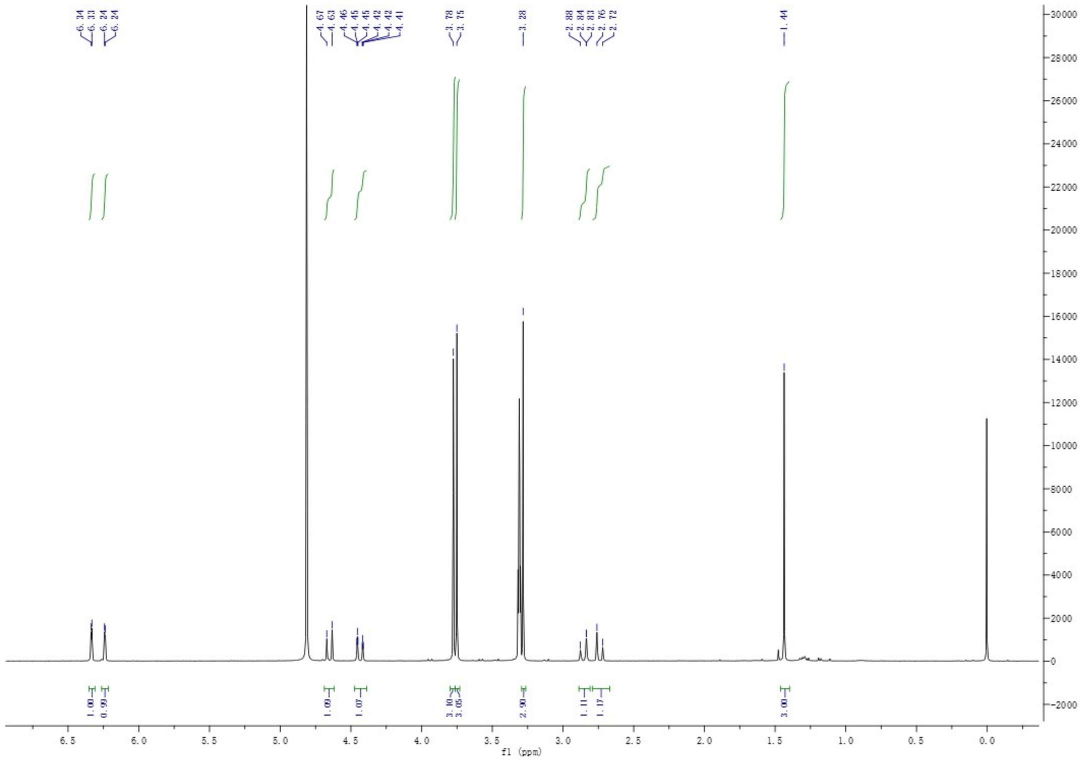

S20. ^{1}H NMR spectrum (400 MHz, CD_3OD) of **3**

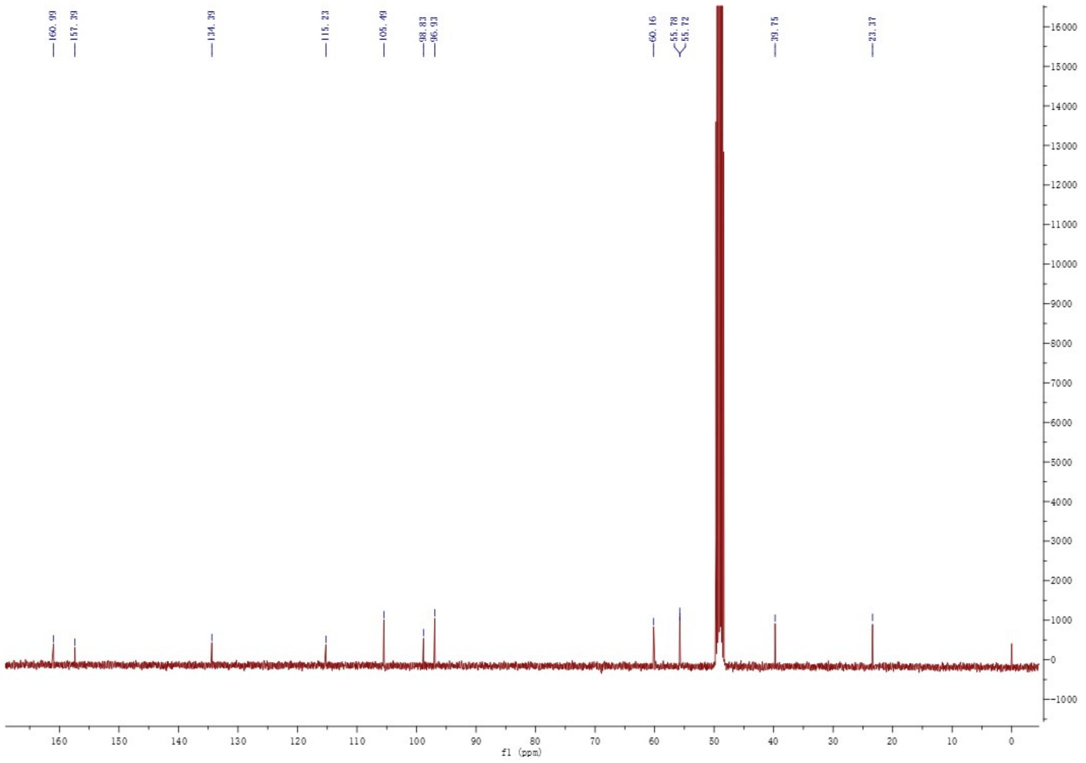

S21. ^{13}C NMR spectrum (100 MHz, CD_3OD) of **3**

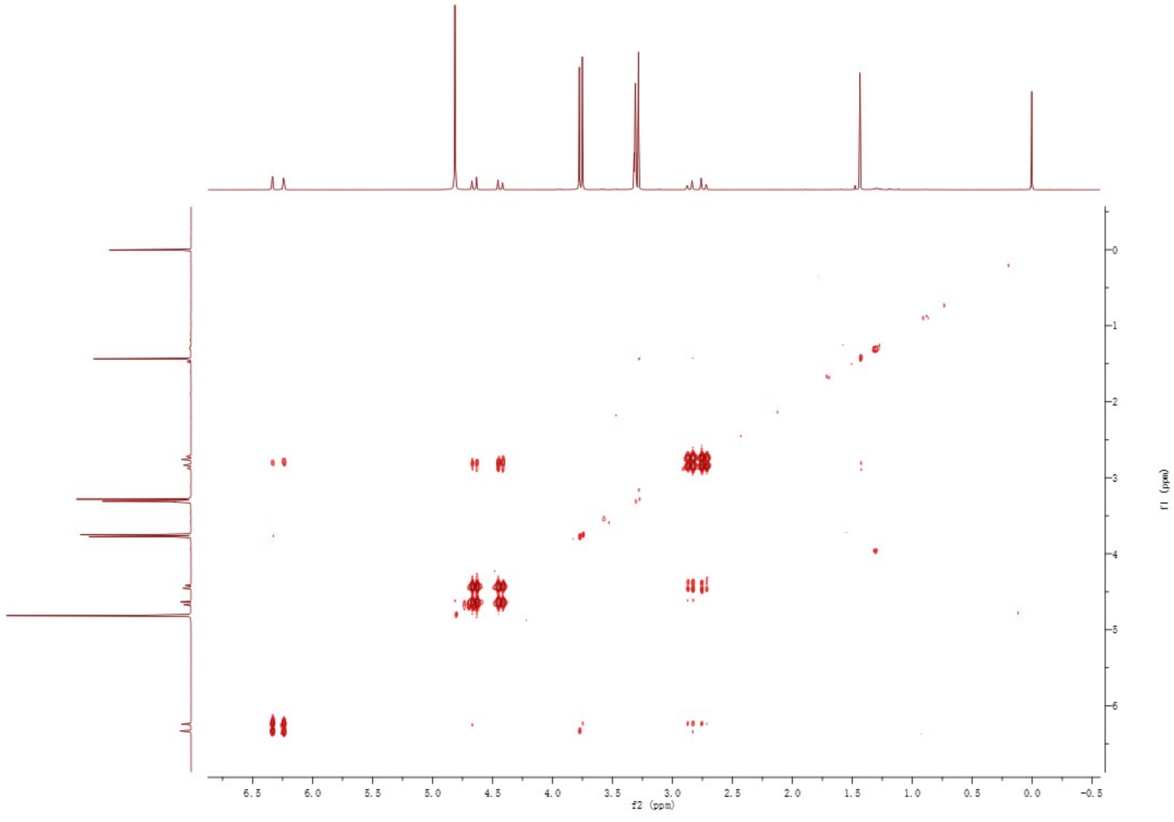

S22. ^{1}H-^{1}H COSY spectrum (CD_3OD) of **3**

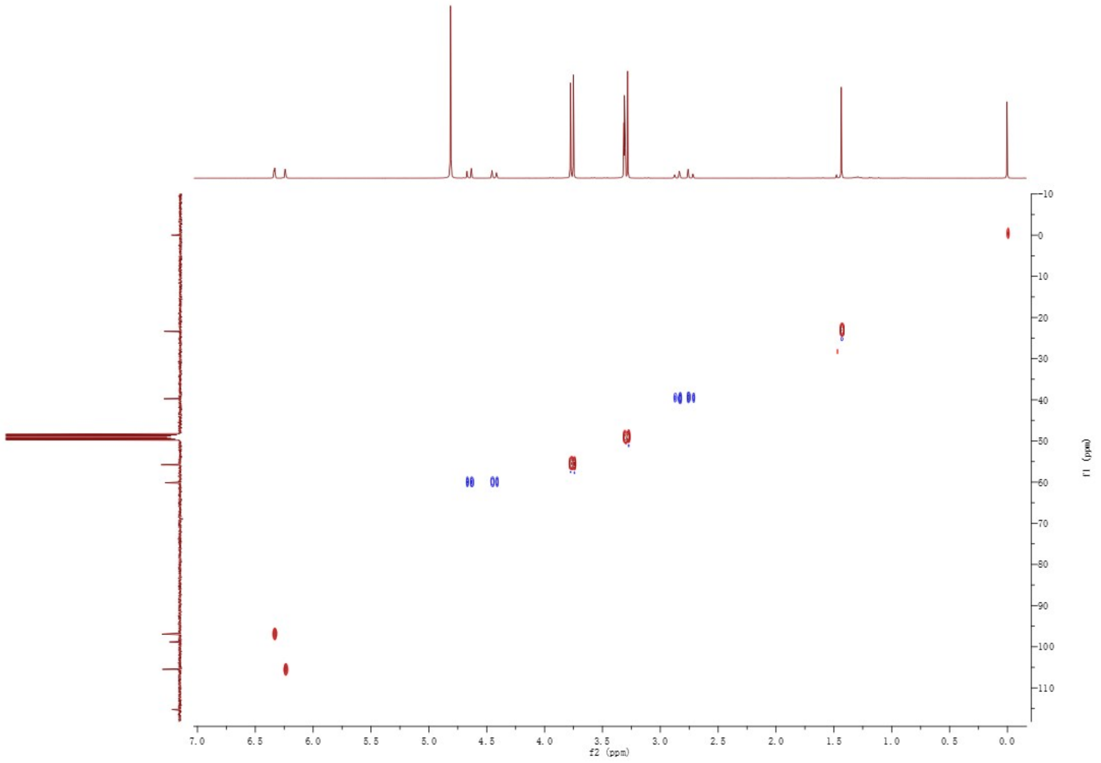

S23. HSQC spectrum (CD_3OD) of **3**

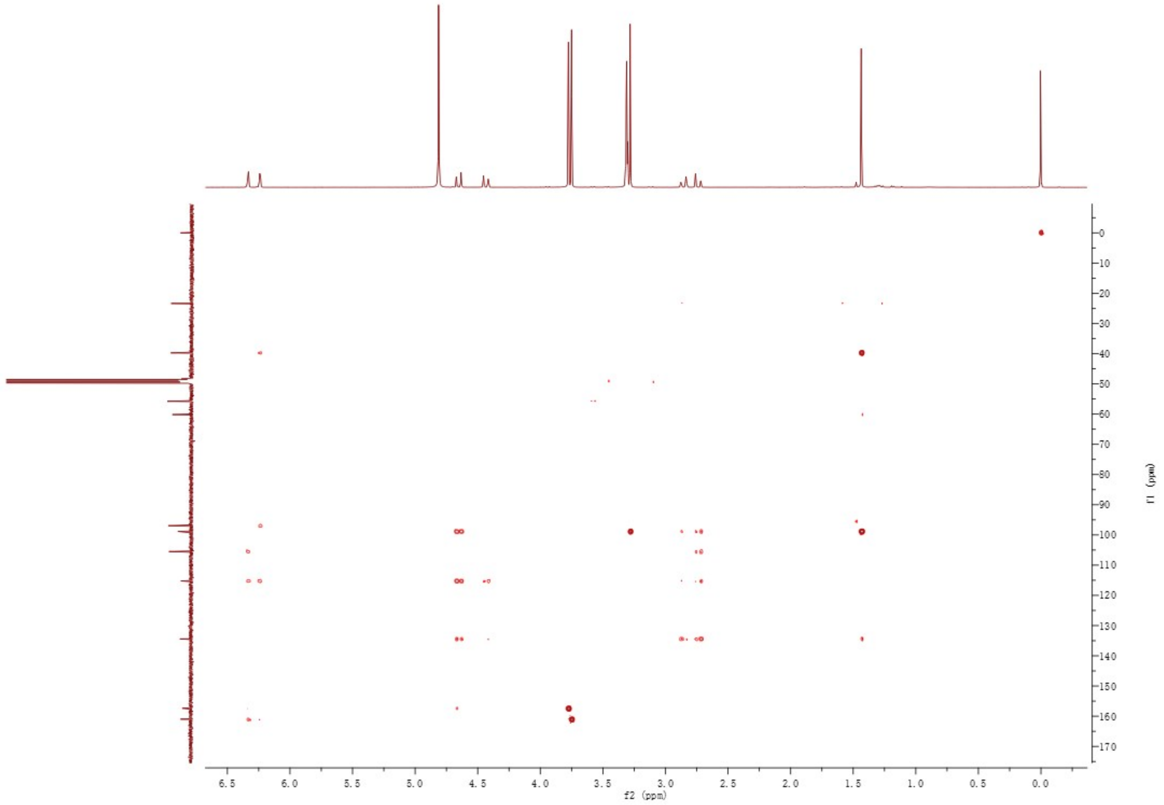

S24. HMBC spectrum (CD_3OD) of **3**

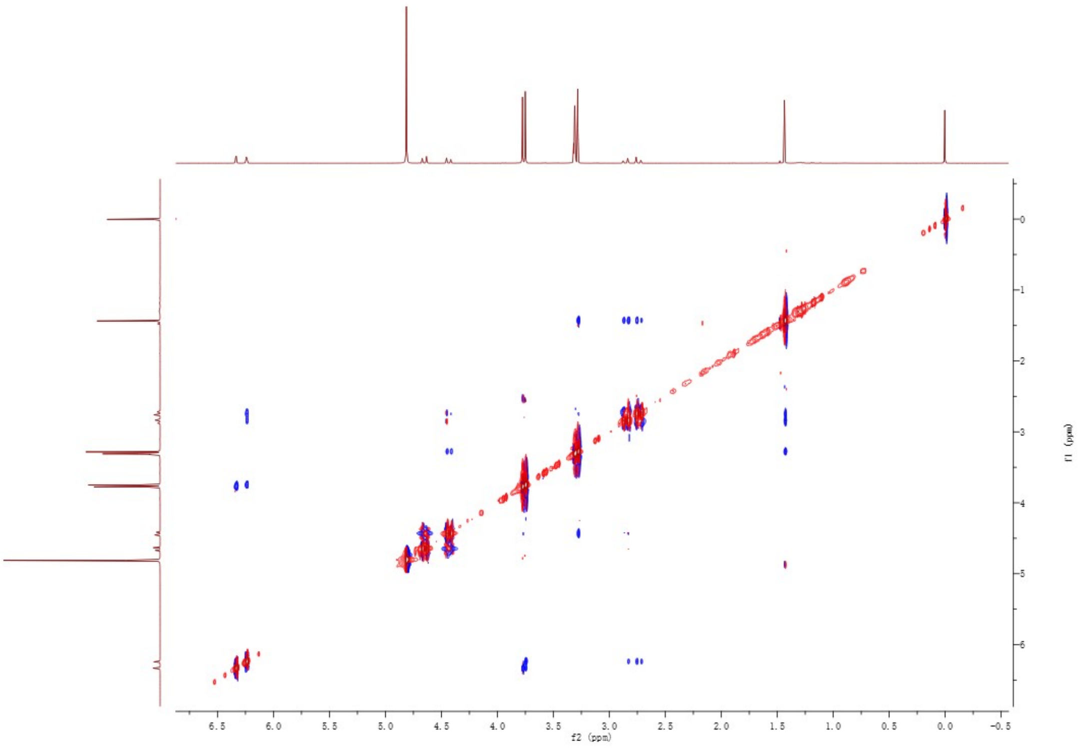

S25. NOESY spectrum (CD_3OD) of **3**
